# Is Vitamin D Deficiency associated with Non Specific Musculoskeletal Pain?

**DOI:** 10.5539/gjhs.v5n1p107

**Published:** 2012-11-11

**Authors:** Mahnaz Abbasi, Sima Hashemipour, Fatemeh Hajmanuchehri, Amir Mohammad Kazemifar

**Affiliations:** 1 Metabolic Diseases Research Center, Qazvin University of Medical Science, Qazvin, Iran

**Keywords:** Vitamin D deficiency, musculoskeletal pain, Vitamin D supplementation, Iran

## Abstract

**Backgrounds:**

Vitamin D deficiency is common worldwide, including Iran. It has been suggested that vitamin D deficiency is associated with non-specific musculoskeletal pain. The aim of this study is evaluation of the association of musculoskeletal pain with vitamin D deficiency and the response of the patients to vitamin D supplementation.

**Materials and Methods:**

sixty two adult patients with chief complaint of musculoskeletal pain were enrolled in the study. Serum concentrations of 25(OH)D, Calcium, Phosphate, Alkaline Phosphatase and PTH were determined. If there was vitamin D deficiency, oral vitamin D supplementation was given. Assessment of pain and its response to therapy was carried out using Visual Assessment Score (VAS). SPSS software version 15.0 was used for statistical analyses.

**Findings:**

Most of the patients (95.4%) had vitamin D deficiency. Pain in 53 patients (85.5%) with responded to the proposed treatment. In responder group post treatment vitamin D concentration was significantly higher than non responder group (60.6±27.6and 39.2±9.6 nmol/l respectively, p<0.01) pretreatment vitamin D and minerals concentrations and pain characteristics did not have significant differences in responder and non responder group.

**Conclusion:**

Treatment with vitamin D can relieve the pain in majority of the patients with vitamin D deficiency. Lack of response can be due to insufficient increase in serum vitamin D concentration. Physiologic differences of gastrointestinal vitamin D absorption, differences of body mass indexes, and noncompliance could be potential causes for this issue. Reassessment of serum 25(OH)D concentration is recommended in nonresponsive patients.

## 1. Introduction

Vitamin D deficiency is common worldwide especially in Asian countries. Recent studies have revealed that it is even prevalent in countries with good sunshine (Alagöl et al., 2000; Sedrani, 1984; Gowami et al., 2000). According to the recent studies prevalence of vitamin D deficeincy is 66-83 percent in Iran (Hashemipour et al., 2004; Azizi, Rais-Zadeh & Mir Said Ghazi, 2000).

Calcium absorption from GI is reduced in vitamin D deficiency. It results in increased secretion of parathyroid hormone and increased activity of osteoclasts. Osteopenia, osteoporosis, and osteomalacia are the end results (Holick, 2003).

Prolonged chronic vitamin D deficiency can result in osteomalacia; moreover mild vitamin D deficiency may produce a variety of musculoskeletal pains such as fibromyalgia-like pain, low back pain, and arthralgia (de Torrenté de la Jara, Pécoud, & Favrat, 2006).

In USA, 9-20% of adults complaint from chronic pains and 89% of them suffer from short or long term disabilities and altered quality of life. Direct and indirect cost of the condition is up to 50 million dollars yearly (Katz, 2002).

Among patients with chronic pain, specific origin of the pain is found only in 25%; for others idiopathic unidentified etiologies should be considered (Plotnikoff & Quigley, 2003).

Some studies have suggested vitamin D deficiency as origin of non-specific musculoskeletal pain including low back pain (Plotnikoff & Quigley, 2003; Lotfi et al., 2007; Heidari et al., 2010).

Current study was conducted to evaluate frequency of vitamin D deficiency among patients with chronic musculoskeletal pain and also clinical response of vitamin D deficient patients to vitamin D supplements. Clinical and lab data of the respondent and non-respondent patients were compared.

## 2. Materials and Methods

Present descriptive analytic study has been performed in Buali hospital, a referral teaching hospital in Qazvin city, Iran. Sixty five adult patients (more than 16 years old) were included in the study. All of them had been referred to rheumatology outpatient clinic with chief complaint of musculoskeletal pain for more than 2 months. If they were taking calcium or multivitamin supplements, their preparations had been discontinued for 2 weeks before starting of the study, patients with any rheumatologic diseases, intervertebral disk diseases, anatomic skeletal malformations, renal disorders, cirrhosis, malabsorption syndrome, and drug history of vitamin D supplements, anticonvulsants and anti-TB drugs were excluded from the study.

Serum samples were taken from the patients for assessment of 25(OH)D, Calcium, Phosphate, Alkaline Phosphatase, and PTH concentrations. Calcium level was determined by calorimetry using a commercial kit (Zist shimi, Iran). Serum vitamin D was determined by ELISA reader awareness and a commercial kit (Euroimmun, Germany). The intraassay and interassay for 25 (OH) D were 3.3% and 6.7%, respectively. If the patients had vitamin D deficiency, they were treated with oral vitamin D3 50000 unit per week for 12 weeks and 1000 mg/day elemental calcium. Three months after end of the treatment they were reassessed for response of their pain to the treatment. Visual Assessment Score (VAS) was used for assessment of the pain severity before or after of the treatment. Serum concentrations of 25(OH) D, Calcium, Phosphate, Alkaline Phosphatase, and PTH were compared in patients who responded and didn’t respond to the treatment.

T-test and Mann whitney were used to compare quantitative parameters with normal and abnormal distribution respectively. Chi-square test was used to compare the relative frequencies. SPSS software version 15.0 was used for statistical analyses. Differences with P value less than 0.05 were considered significant.

## 3. Results

Frequency distribution of age and biochemical parameters of the patients is demonstrated in Table 1. Most of the patients (95.4%) had vitamin D deficiency (serum 25 (OH) D concentration less than 50 nmol/l). The deficiency was severe (serum 25 (OH) Dconcentration less than 25 nmol/l) in 74.2% of them.

**Table 1:** Basic characteristics of participates in study

	Total
Age (year)	35.4 ±12.4
calcium (mg/dl)	9.1 ± 0.4
PTH (pmol/l)	47.6 ±45.1
Phosphate(mg/dl)	3.4 ± 0.6
Alkaline phosphatase (IU/l)	186.7±104.3
(25OH)D (nmol/l)	20.6 ± 10.0
(25OH)D <25 nmol/l	74.2%
(25OH)D< 12 nmol/l	24.0%

Fifty three patients (85.5%) with vitamin D deficiency responded to the proposed treatment and their VAS scores diminished more than 60%. In 47 (75.8%) patients the pain subsided completely. The patients with vitamin D deficiency were divided into respondent and non-respondent groups according to response of their pain to the proposed treatment. As it is shown in Table 2 serum concentrations of 25 (OH) D, Calcium, Phosphate, Alkaline Phosphatase, and PTH were not significantly different between two groups. However serum concentration of 25 (OH) D was significantly different after the proposed treatment (60.6±27.6nmol/l vs. 39.2± 9.6 nmol/l, P value < 0.01). Rising of serum vitamin D in responder group was about 2.5 times higher than non responder group.

**Table 2:** Comparison of age and laboratory parameters between pain responders and non-responders

	responders	non-responders	P value
Age (year)	35.5±12.8	35.4±13.2	Non sig
Calcium (mg/dl)	9.1 ± 0.4	9.1±0.3	Non sig
Hypocalcemia (%)	16.9%	22.2%	Non sig
PTH (pmol/l)	49.7±48.6	37.3±30.7	Non sig
Phosphate (mg/dl)	3.4±0.6	3.7±0.7	Non sig
(25OH)D (nmol/l)	20.7±10.2	22.8±10.2	Non sig
(25OH)D <25 (nmol/l)	77.3%	55.5%	Non sig
(25OH)D< 12 (nmol/l)	24.5%	22.2%	Non sig
(25OH)D after treatment	60.6±27.6	39.2±9.6	P< 0/01
change of 25(OH)D(nmol/l)	40.3±29.0	16.4±12.0	P< 0/05

Characteristics of the pain were the same in the groups. The most common sites of the pain were lower limb (in 73.6% and 100% of the patients in respondent and non-respondent groups respectively) and upper limb (in 49.1% and 55.6% of the patients in respondent and non-respondent groups respectively) (Table 3).

**Table 3:** Comparison of nature of pain between vitamin D therapy responder and non-responder groups

		responders	non-responders	P value
Location of pain	Bone	92.5%	100%	Non sig
	muscle	13.2%	11.1%	Non sig
Anatomic site of the pain	Lower limbs	73.6%	100%	Non sig
	Upper limb	49.1%	55.6%	Non sig
	Low back	32.1%	44.4%	Non sig
	shoulder	22.6%	33.3%	Non sig
Presence of tenderness		64.2%	77.8%	Non sig

## 4. Discussion

Results of current study showed that 95.4% of patients with musculoskeletal pain suffered from vitamin D deficiency and the pain responded to vitamin D supplementation in most of the patients. In patients who respond to vitamin D supplementation, more notable rise in serum concentrations of 25(OH)D was detected.

Relationship between vitamin D deficiency and musculoskeletal pain has been drawn attention of many investigators. Gregory and his coworkers studied 150 patients with musculoskeletal pain. They showed that 93% of them had vitamin D deficiency (Plotnikoff & Quigley, 2003). In study conducted by Heidari et al. (2010) in Iran on 276 patients with musculoskeletal pain, 63.4% of the patients had vitamin D deficiency. Study of Al Faraj and Al Mutairi (2003) demonstrated that 83% of patients with low back pain have vitamin D deficiency. There are other similar studies that have suggested association between above mentioned variables (De Torrenté de la Jara, Pécoud, & Favrat, 2004; Reginato et al., 1999; Mouyis et al., 2008; Bartley, 2008); however a few studies have found no association between vitamin D deficiency and musculoskeletal pain (Block, 2004; Warner & Arnspiger, 2008).

Vitamin D is essential for GI absorption of calcium. Vitamin D deficiency results in increased secretion of parathyroid hormone (PTH) which in turn leads to greater than before activity of osteoclasts in bone. Phosphaturic property of PTH with diminished absorption of calcium from GI may reduce calcium-phosphate interaction and consequently bone mineralization. Increased activity of osteoclasts and osteoblasts increases bone matrix with low mineralization. The resultant matrix would absorb water; as a result subperiosteal space becomes edematous. The event may produce bone pain (Holick, 2003).

Present study confirmed that vitamin D supplementation can relieve the pain in majority of the patients; finding that agree with study of Al Faraj (Al Faraj & Al Mutairi, 2003). Mean rise in serum 25 (OH) D concentrations was 2.5 fold higher in respondent group compared to non-respondent group. Ryan has reported variable rise in serum 25 (OH) D concentrations after vitamin D supplementation too (Ryan, 2007). It has suggested that BMI (body mass index) are determinate of extent of rise in serum 25(OH)D concentration after vitamin D supplementation; however age of the patient was comparable between 2 groups in current study. Study of Ryan has found no association between age of the patients and extent of rise in serum 25(OH) D cholecalciferol concentration (Ryan, 2007). Body mass index and poor compliance are other factors which can influence serum vitamin D level after treatment of vitamin D deficiency.

Regardless of the cause of inadequate rise of serum vitamin D to treatment in some patients, results of this study point out the necessity of repeated measurement of serum vitamin D in patients with vitamin D deficiency and musculoskeletal pain whose their pain does not response to oral vitamin D supplementation. In these cases retreatment with vitamin D is warranted if inadequate rise of serum vitamin D is detected.

Some authors have suggested some specific character for the pain associated with vitamin D deficiency (Holick, 2003). They believe that the pain is usually sensed on the bone or muscle. In present study 93.5 % and 12.9% of patients were complaining from bone and muscle pains respectively. Also it is believed that bone tenderness at sternum, tibia, radius or ulna is common presentation of vitamin D deficiency. 66.1% the patients in present study had bone tenderness, more commonly in lower limbs.

The most common anatomic locations for pain were upper and lower limbs respectively in current study. Also some patients had pain in low back and shoulder areas. In study of Reginato and his colleagues the most common locations for pain were thoracic and lumbar spines, shoulder, ribs and pelvic areas (Reginato, et al., 1999). Study of Heidari et al. conducted in Iran showed that the most common site for pain is lower limbs (Heidari, et al., 2010). Nature of pain had not distinction among 2 studied groups in present study. It can be concluded that the nature of pain has not prognostic significance.

There were some limitations in present study. It was better to be carried out as a randomized controlled trial; however administrative difficulties precluded this. BMI was not measured in current study. Non compliance to treatment is another potential cause of insufficient rise of serum vitamin D that was not evaluate in our study.

In summary, findings of present study revealed that vitamin D deficiency is widespread among Iranian population and it may be associated with musculoskeletal pain which relieve with vitamin D supplementation; in pain nonresponders reassessment of serum 25(OH) D concentrations is recommended.

## References

[ref1] Al Faraj S, Al Mutairi K (2003). Vitamin D deficiency and chronic low back pain in Saudi Arabia. Spine.

[ref2] Alagöl F, Shihadeh Y, Boztepe H, Tanakol R, Yarman S, Azizlerli H, Sandalci O (2000). Sunlight exposure and vitamin D deficiency in Turkish women. J Endocrinol Invest.

[ref3] Azizi F, Rais-Zadeh F, Mir Said Ghazi A (2000). Vitamin D deficiency in a group of Tehran Population. Res Med.

[ref4] Bartley J (2008). Prevalence of vitamin D deficiency among patients attending a multidisciplinary tertiary pain clinic. N Z Med J.

[ref5] Block S. R (2004). Vitamin D Deficiency is not associated with nonspecific musculoskeletal pain syndromes including fibromyalgia. Mayo Clin Proc.

[ref6] de Torrentéde la Jara G, Pécoud A, Favrat B (2004). Musculoskeletal pain in female asylum seekers and hypovitaminosis D3. BMJ.

[ref7] de Torrentéde la Jara G, Pécoud A, Favrat B (2006). Female asylum seekers with musculoskeletal pain: the importance of diagnosis and treatment of hypovitaminosis D. BMC Fam Pract.

[ref8] Goswami R, Gupta N, Goswami D, Marwaha R. K, Tandon N, Kochupillai N (2000). Prevalence and significance of low 25-hydroxyvitamin D concentrations in healthy subjects in Delhi. Am J Clin Nutr.

[ref9] Hashemipour S, Larijani B, Adibi H, Javadi E, Sedaghat M, Pajouhi M, Booya F (2004). Vitamin D deficiency and causative factors in the population of Tehran. BMC Public Health.

[ref10] Heidari B, Shirvani J. S, Firouzjahi A, Heidari P, Hajian-Tilaki K. O (2010). Association between nonspecific skeletal pain and vitamin D deficiency. Int J Rheum Dis.

[ref11] Holick M. F (2003). Vitamin D deficiency: what a pain it is. Mayo Clin Proc.

[ref12] Holick M. F (2003). Vitamin D: A millennium perspective. J Cell Biochem.

[ref13] Katz W. A (2002). Musculoskeletal pain and its socioeconomic implications. Clin Rheumatol.

[ref14] Lotfi A, Abdel-Nasser A. M, Hamdy A, Omran A. A, El-Rehany M. A (2007). Hypovitaminosis D in female patients with chronic low back pain. Clin Rheumatol.

[ref15] Mouyis M, Ostor A. J, Crisp A. J, Ginawi A, Halsall D. J, Shenker N, Poole K. E (2008). Hypovitaminosis D among rheumatology outpatients in clinical practice. Rheumatology.

[ref16] Plotnikoff G. A, Quigley J. M (2003). Prevalence of severe hypovitaminosis D in patients with persistent, nonspecific musculoskeletal pain. Mayo Clin Proc.

[ref17] Reginato A. J, Falasca G. F, Pappu R, McKnight B, Agha A (1999). Musculoskeletal manifestations of osteomalacia: report of 26 cases and literature review. Semin Arthritis Rheum.

[ref18] Ryan P. J (2007). Vitamin D therapy in clinical practice. One dose does not fit all. Int J Clin Pract.

[ref19] Sedrani S. H (1984). Low 25-hydroxyvitamin D and normal serum calcium concentrations in Saudi Arabia: Riyadh region. Ann Nutr Metab.

[ref20] Warner A. E, Arnspiger S. A (2008). Diffuse musculoskeletal pain is not associated with low vitamin D levels or improved by treatment with vitamin D. J Clin Rheumatol.

